# Compound effects of aging and experimental FSGS on glomerular epithelial cells

**DOI:** 10.18632/aging.101176

**Published:** 2017-02-17

**Authors:** Remington R.S Schneider, Diana G. Eng, J. Nathan Kutz, Mariya T. Sweetwyne, Jeffrey W. Pippin, Stuart J. Shankland

**Affiliations:** ^1^ Division of Nephrology, University of Washington, Seattle, WA 98109, USA; ^2^ Department of Applied Mathematics, University of Washington, Seattle, WA 98109, USA

**Keywords:** kidney disease, glomerulus, parietal epithelial cell, podocyte, epithelial to mesenchymal transition, Collagen IV

## Abstract

Advanced age portends a poorer prognosis in FSGS. To understand the impact of age on glomerular podocytes and parietal epithelial cells (PECs), experimental FSGS was induced in 3m-old mice (20-year old human age) and 27m-old mice (78-year old human age) by abruptly depleting podocytes with a cytopathic anti-podocyte antibody. Despite similar binding of the disease-inducing antibody, podocyte density was lower in aged FSGS mice compared to young FSGS mice. Activated PEC density was higher in aged versus young FSGS mice, as was the percentage of total activated PECs. Additionally, the percentage of glomeruli containing PECs with evidence of phosphorylated ERK and EMT was higher in aged FSGS mice. Extracellular matrix, measured by collagen IV and silver staining, was higher in aged FSGS mice along Bowman's capsule. However, collagen IV accumulation in the glomerular tufts alone and in glomeruli with both tuft and Bowman's capsule accumulation were similar in young FSGS and aged FSGS mice. Thus, the major difference in collagen IV staining in FSGS was along Bowman's capsule in aged mice. The significant differences in podocytes, PECs and extracellular matrixaccumulation between young mice and old mice with FSGS might explain the differences in outcomes in FSGS based on age.

## INTRODUCTION

Age is a multi-faceted contributor to morbidity and mortality. With recent advances in science, medicine and standards of living, people are living longer. However, with increasing age, humans begin to lose the working capabilities of several organs. One well-documented example is the kidney [1]. The prevalence of impaired kidney function is 15% higher in elderly individuals over 70 years of age compared younger adults [1, 2]. In addition, chronic kidney disease is disproportionately more common in persons over 65 years old [1, 3].

As they age, people lose kidney tissue and the number of nephrons decreases [4-7]. Renal plasma flow decreases in the kidney's outer cortex and to a lesser extent, in the medulla [8]. This is primarily due to the hardening of blood vessels, thereby constricting the filtration capabilities of nephrons [9]. Glomerular filtration rate (GFR) declines by 10% per year after 40 years of age, at a loss of about 0.7-0.9 ml/min/year [10]. The relatively larger decrease in renal plasma flow over GFR contributes to an increasing filtration fraction [9]. In addition to these age-related functional changes, structural abnormalities to the glomerulus have been documented in aged mice, rats and humans [2, 7, 11, 12]. Glomerular size increases with age, especially in the juxta-medullary region of the kidney [2, 13, 14]. Glomerulosclerosis also progresses with advanced age [2, 8, 9]. In addition, both glomerular epithelial cell types, i.e. podocytes and parietal epithelial cells (PECs), are adversely affected by advanced age, including a decrease in both their cell number and density [2, 15].

Studies have shown that for most clinical glomerular diseases, renal outcomes are worse in aged individuals compared to younger cohorts with a similar disease [2, 8]. However, the mechanisms underlying these age-related differences are not well understood. Although co-morbid conditions such as hypertension might contribute to worse kidney outcomes in the older population, different responses by both glomerular epithelial cell types to glomerular disease might underlie differences between young and aged outcomes. Accordingly, this study examines if and how PECs and podocytes might differ in their responses in young mice (3m-old, equivalent to human aged 20 years) and in aged mice (24m-old, equivalent to human aged 70 years) with experimental focal segmental glomerulo-sclerosis (FSGS) [16].

## RESULTS

### Sheep IgG staining was similar in young and aged animals

**Figure 1:**
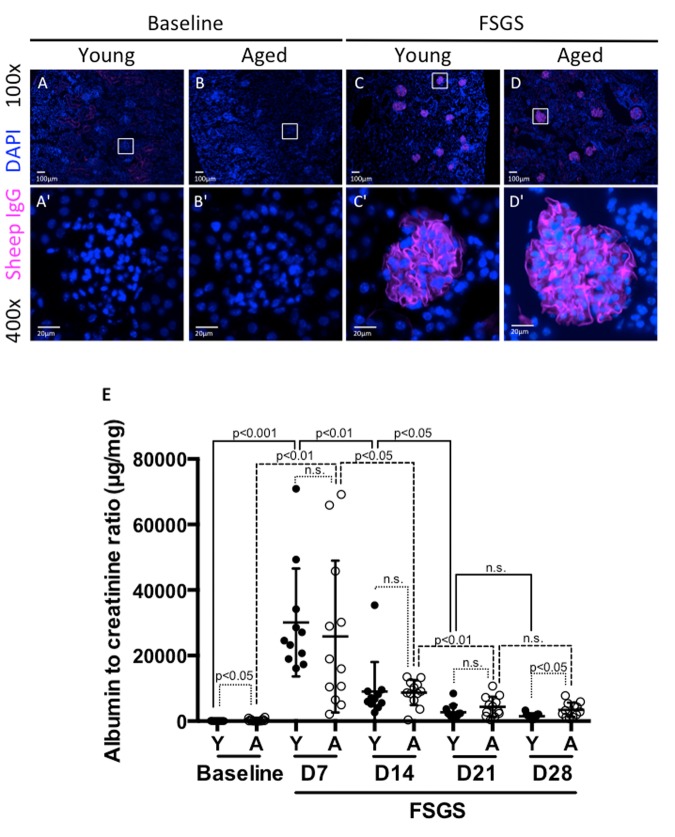
Albuminuria was higher in aged mice at baseline and in FSGS

Experimental FSGS was induced in young and aged mixed gender groups of mice with a cytopathic sheep anti-glomerular antibody [17, 18]. Podocyte deposition was confirmed by sheep IgG staining, as we have previously reported [17, 18]. Sheep IgG staining was absent in all baseline tissues as expected, but was present and indistinguishable in both young and aged mice given sheep anti-glomerular antibody (Figure 1A-D). This result shows that in both age groups, the antibody bound exclusively to the glomerular tuft within the kidney, and the results described below were likely not due to differential affinity of the antibody based on age.

(**A-D**) Sheep IgG staining. Sheep IgG staining confirmed the equal distribution of anti-glomerular antibody in the glomerular tuft in both young (Y) and aged (A) mice. Images were taken at 100x. (**A'-D'**) 400x close-up images of glomeruli from (**A-D**). Sheep IgG deposition (purple color) was not seen in baseline mice as expected (**A,B,A',B'**). Animals given FSGS with the anti-glomerular antibody showed sheep IgG deposition in the glomerular tufts (**C,D,C’,D'**). (**E**) Albumin to creatinine ratios (*ACR)*. ACRs (μg/mg) for young mice (black circles), and aged mice (white circles), at baseline prior to FSGS and at days 7, 14, 21 and 28 post-FSGS. ACR increased acutely for both young and aged animals to peak at D7, followed by gradual recovery for 21 days. Aged mice started with higher ACR at baseline and finished with significantly higher ACR at D28.

### Urine analysis

We have previously demonstrated that this experimental model is characterized by acute injury, which peaks early, with substantial recovery when animals are followed for 28 days [17, 19, 20]. We assessed urine albumin-to-creatinine ratio (ACR-μg/mg) weekly from baseline to D28. Aged baseline mice had higher ACR compared to young baseline mice (302±384 aged vs. 38±17 young, p<0.05) (Figure 1E).

There was a significant increase in ACR by D7 of FSGS in both young and aged mice (38±17 to 30075±16468, p<0.001 vs. young baseline; 302±384 to 25800±23138, p<0.01 vs. aged baseline). When calculated as a fold-increase from baseline to D7, young mice experienced a 782 fold increase, and aged mice experienced an 85 fold increase. Despite the large increases in both groups, there was no significant difference between young vs. aged groups (p=0.098 vs. aged baseline-D7 fold change). As we have previously published [19, 20], there was a significant decrease in ACR between D7 and D14 for both young (30075±16468 to 9056±8692, p<0.01 vs. young D7) and aged mice (25800±23138 to 8726±3848, p<0.05 vs. aged D7). While young mice experienced a 3.3 fold decrease, aged mice experienced a 3.0 fold decrease from D7 to D14. However, there was no significant difference between young and aged groups (p=0.567 vs. aged D7-D14 fold change).

Between D14 and D21, both young and aged mice experienced a further decrease in ACR (9056±8692 to 2726±2226 p<0.05 vs. young D14; 8726±3848 to 4348±3092 p<0.01 vs. aged D14). Over the time period from D14 to D21, young mice experienced a 3.3 fold decrease, while aged mice showed less FSGS resolution with only a 2 fold decrease. The fold changes were not statistically significant between young and aged groups. (p=0.117 vs. aged D21-D14 fold change).

Between D21 and D28, both groups experienced decreases in ACR that were not statistically significant (2726±2226 to 1497±842, p=0.111 vs. young D21; 4348±3092 to 3457±2167, p=0.423 vs. aged D21). When calculated as a fold decrease, young mice experienced a 1.8 fold decrease and aged mice experienced a 1.3 fold decrease. By D28 of FSGS, aged mice maintained a significantly higher ACR than young mice (3457±2167 vs. 1497±842, p<0.05).

Taken together, these results show that aged mice have a higher ACR at the beginning and end of the study.

While young FSGS mice have more substantial fold decreases in weekly ACR measurements than aged mice (i.e. recovery), these differences in fold decreases did not reach our selected level of statistical significance due to the variability between animals, and small study cohorts, which are not unique to this study, especially when dealing with aged animals [21].

### Depletion in podocyte density was more severe in aged mice with FSGS

**Figure 2:**
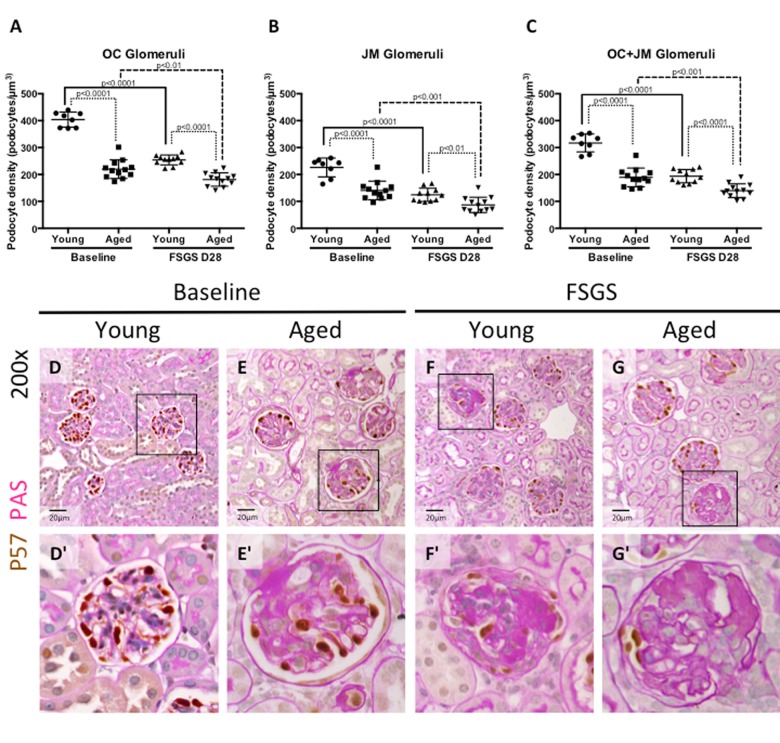
Podocyte density was lower at baseline in aged mice, and in aged mice with FSGS (**A-C**) Quantification of podocyte density. Graphs A and B show the average podocyte density in podocytes per glomerular volume (μm^3^) for individual animals in OC and JM glomeruli respectively. Graph C shows podocyte density for individual animals when OC and JM glomeruli are combined, which serves as a representation of the entire section. Podocyte density was lower in aged baseline mice than young baseline mice in glomeruli of the OC (**A**), JM (**B**), and when combined (**C**). Aged FSGS mice also had lower podocyte density in OC (**A**), JM (**B**), and combined (**C**) glomeruli than young FSGS mice, despite young mice experiencing a larger magnitude of podocyte depletion with FSGS. (**D-G**) PAS/p57 double staining. Representative images of glomeruli at 20x magnification, with higher magnifications shown in D’-G’ of the glomerulus marked by solid black square. Podocytes were identified by p57^+^ staining (brown color, nuclear) against the pink PAS counterstain.

Secondary FSGS is characterized by reduced podocyte density [19]. Accordingly, podocyte density (defined as the number of podocytes per glomerular volume in μm^3^) was calculated to account for age-related changes in glomerular size. Podocyte density was analyzed at baseline and with FSGS by kidney region, by separating glomeruli of the outer cortex (OC) from glomeruli of the juxta-medulla (JM), as previously described [2]. Combined podocyte density was defined as the sum of OC and JM glomeruli. The following summarizes the podocyte density results:

***Young baseline versus aged baseline*** (i.e. prior to disease) (Figure 2A, B, C, D, D’, E, E’): In OC glomeruli, podocyte density was 45.61% lower in aged baseline mice compared to young baseline mice (219.7±35 vs. 403.9±28, p<0.0001 aged baseline vs. young baseline). In JM glomeruli, podocyte density was 38.05% lower compared in aged mice (140.7±35 vs. 226.0±35, p<0.0001 aged baseline vs. young baseline). Overall, aged baseline mice had 40.33% lower combined OC and JM podocyte density compared to young baseline mice (189.2±35 vs. 317.1±33, p<0.0001 aged baseline vs. young baseline).

***Young mice with FSGS*** (Figure 2A, B, C, D, D’, F, F’): Following the administration of the cytotoxic antibody to induce podocyte depletion in young mice, podocyte density decreased by 37.09% in OC glomeruli (254.1±18 vs. 403.9±28, p<0.0001 young FSGS vs. young baseline), by 44.96% in JM glomeruli (124.4±25 vs. 226.0±35 p<0.0001 young FSGS vs. young baseline) and by 38.95% in combined OC and JM glomeruli from baseline (193.6±25 vs. 317.1±33, p<0.0001 young FSGS vs. young baseline), at FSGS D28.

***Aged mice with FSGS*** (Figure 2A, B, C, E, E’, G, G’): Following podocyte injury in aged mice, podocyte density decreased by 17.52% in OC glomeruli (181.2±25 vs. 219.7±35 p<0.01 aged FSGS vs. aged baseline), by 38.31% in JM glomeruli (86.8±28 vs. 140.7±35, p<0.001 aged FSGS vs. aged baseline) and by 26.16% in combined OC and JM glomeruli compared to aged baseline (139.7±26 vs. 189.2±35, p<0.001 aged FSGS vs. aged baseline).

***Young FSGS versus aged FSGS*** (Figure 2A, B, C, F, F’, G, G’): In aged FSGS mice compared to young FSGS mice, podocyte density was 28.69% lower in OC glomeruli (181.2±25 vs. 254.1±18 p<0.0001 aged FSGS vs. young FSGS), 30.23% lower in JM glomeruli (86.8±28 vs. 124.4±25 p<0.01 aged FSGS vs. young FSGS) and 27.84% lower in combined OC and JM glomeruli (139.7± 26 vs. 193.6±25, p<0.0001 aged FSGS vs. young FSGS).

Taken together, these results show that aged baseline mice have a lower podocyte density in glomeruli in the OC, JM, and combined OC and JM compared to young baseline mice. Although podocyte density decreased to a larger degree from baseline in younger mice with FSGS, podocyte density was still lower in aged FSGS mice in OC, JM and overall glomeruli.

### Despite being lower at baseline, PEC density increas-ed more in aged FSGS mice than young FSGS mice

**Figure 3:**
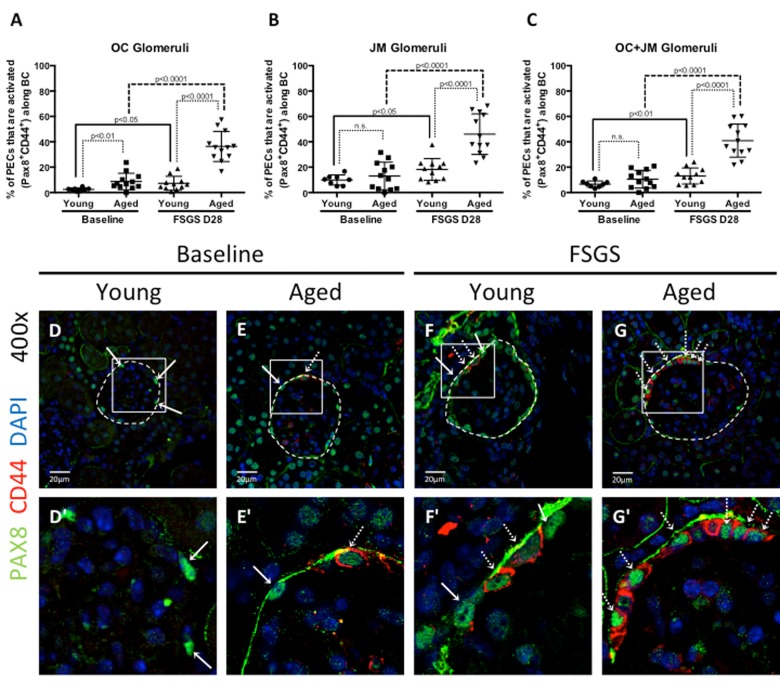
Parietal epithelial cell (PEC) activation was highest in aged FSGS mice PECs were identified by PAX8 staining (green color, nuclear), and the subset of PECs undergoing activation were identified by CD44 staining (red color, cytoplasmic). (**A-C**) Quantitation of the percentage of PECs that are activated. (**A**) In outer cortical (OC) glomeruli, the percentage of PECs that were activated (PAX8^+^CD44^+^) along Bowman's capsule (BC) was higher in aged mice at baseline and in FSGS. (**B**) In juxta-medullary (JM) glomeruli, the percentage of activated PECs were similar at baseline, but higher in aged FSGS mice compared to young FSGS mice. (**C**) When OC and JM glomeruli were combined, aged FSGS mice had the highest percentage of PECs that were activated. (**D-G**) Representative images of Pax8 and CD44 staining on BC. Images taken at 400x by confocal microscopy for PAX8 (green, nuclear), CD44 (red, cytoplasmic) and DAPI (blue, nuclear) staining. (**D’-G’**) Higher magnification of the white square shown above. Solid arrow shows examples of PAX8 staining; dashed arrow shows examples of CD44 below (**D’-G’**). As shown in the above graphs, the percentage of PECs along Bowman's capsule that are activated was higher in aged baseline mice, and increased further at D28 of FSGS in aged mice.

***Young baseline versus aged baseline***: To better understand the PEC responses in FSGS with advanced age, we began by measuring PEC density. PECs were identified by PAX8^+^ staining (Figure 3) along Bowman's capsule, and because of the potential for age-related changes in glomerular size, Bowman's capsule length (nm) was also measured to generate PEC density measures (Table 1). PEC density was similar in OC glomeruli between young baseline and aged baseline mice (19.77±1.80 vs. 20.30±2.25, p=0.587 aged baseline vs. young baseline), but was lower in JM glomeruli in aged baseline mice (19.66±3.30 vs. 23.85±1.84, p<0.001 aged baseline vs. young baseline). PEC density in combined OC and JM glomeruli was also lower in aged baseline mice (19.45±1.31 vs. 22.09±1.62 p<0.01 aged baseline vs. young baseline).

**Table 1:** PEC and activated PEC density

		Young			Aged			Fold increase and significance in aged mice compared to young mice		
		OC	JM	OC+JM	OC	JM	OC+JM	OC	JM	OC+JM
**PEC density (total pax8 #/BC nm)**	Baseline	20.30 ±2.25	23.85±1.84	22.09±1.62	19.77±1.80	19.66±3.30	19.45±1.31	0.97 fold p=0.587	0.82 fold p<0.01	0.88 fold p<0.01
	FSGS D28	23.33±2.97	27.35±6.79	25.31±3.15	26.99±2.24	26.18±3.92	26.61±2.80	1.16 fold p<0.01	0.96 fold p=0.622	1.05 fold p=0.309
	Fold increase and significance in FSGS from baseline	1.15 fold p<0.05	1.15 fold p=0.129	1.15 fold p<0.05	1.37 fold p<0.0001	1.33 fold p<0.001	1.37 fold p<0.0001			
**Activated PEC density along Bowman's capsule (Activated PEC #/BC nm)**	Baseline	0.50±0.23	1.95±0.95	1.26±0.58	1.64±1.29	2.21±1.82	1.88±1.23	3.28 fold p<0.05	1.13 fold p=0.682	1.49 fold p=0.147
	FSGS D28	1.82 ±1.45	4.97±3.39	3.96±4.07	9.97±3.71	12.43±4.75	11.43±3.78	5.48 fold p<0.0001	2.50 fold p<0.001	2.89 fold p<0.001
	Fold increase and significance in FSGS from baseline	3.64 fold p<0.05	2.55 fold p<0.05	3.14 fold p=0.054	6.08 fold p<0.0001	5.62 fold p<0.0001	6.08 fold p<0.0001			
**% of PECs that are activated**	Baseline	2.49±1.16%	9.89±3.95%	6.66±2.47%	8.73±6.51%	13.06±10.87%	10.52±7.01%	3.51 fold p<0.01	1.32 fold p=0.372	1.58 fold p=0.100
	FSGS D28	7.30±5.47%	18.22±8.61%	13.06±6.31%	36.34±11.84%	46.03±16.00%	40.91±13.05%	4.98 fold p<0.0001	2.53 fold p<0.0001	3.13 fold p<0.0001
	Fold increase and significance in FSGS from baseline	2.93 fold p<0.05	1.84 fold p<0.05	1.96 fold p<0.01	4.16 fold p<0.0001	3.52 fold p<0.0001	3.89 fold p<0.0001			

Labeled in bold, PEC density (row 1), activated PEC density (row 2), and the percent of PECs that are activated (row 3) are given for young and aged mice. For both young and aged mice, values are given for OC, JM, and combined OC and JM. One important note is that the OC+JM value is not necessarily the average of the OC and JM region values. Rather, the combined OC+JM value is the average of 40 OC and 30 JM (when possible) glomeruli together as roughly 70 representative glomeruli of the total section. Data are represented as mean±SD. Fold increases (≥1) from baseline to FSGS, and also between young and aged mice at baseline and FSGS, are given below and to the right side of the table respectively. A fold increase of less than 1 implies a fold decrease. Significance is given in the same box as fold change. OC - outer cortex; JM - juxta-medullary; SD - standard deviation.

***Young FSGS versus aged FSGS***: In OC glomeruli at D28 of FSGS, PEC density increased significantly in both young FSGS mice (23.33±2.97 vs. 20.30±2.25 p<0.05 young FSGS vs. young baseline) and in aged FSGS mice (26.99±2.24 vs. 19.77±1.80, p<0.0001 aged FSGS vs. aged baseline). In JM glomeruli at D28 FSGS, PEC density increased in aged mice (26.18±3.92 vs. 19.66±3.30, p<0.001 aged FSGS vs. aged baseline), but not in JM glomeruli of young FSGS mice (27.35±6.79 vs. 3.85±1.84, p=0.129 young FSGS vs. young baseline). When OC and JM glomeruli were combined, PEC density increased 1.15 fold in young FSGS mice (p<0.05 vs. young baseline), and 1.37 fold in aged FSGS mice (p<0.0001 vs. aged baseline).

These results show that PEC density was lower in aged baseline mice compared to young baseline mice. However, because PEC density increased more in aged FSGS mice, there were no differences in the final PEC density by D28 FSGS.

### Activation of PECs along Bowman's capsule was higher in aged mice with FSGS

Because activation of PECs is increasingly recognized as a major mechanism underlying segmental glomerular scarring in FSGS [22-25], we next asked if PEC activation in disease was impacted by advanced age. The density of activated PECs on Bowman's capsule were defined as cells lining Bowman's capsule that double-stained for Pax8 and CD44 (Pax8^+^CD44^+^) (Figure 3), using Bowman's capsule length (nm) as the denominator. Table 1 reports activated PEC density.

***PEC activation at baseline***: At baseline in OC glomeruli, the density of activated PECs was significantly higher (3.28 fold increase) in aged mice compared to young mice (1.64±1.29 vs. 0.50±0.23, p<0.05 aged baseline vs. young baseline). However, there was only a 1.13 fold difference in the density of activated PECs in JM glomeruli (2.21±1.82vs. 1.95±0.95, p=0.682 aged baseline vs. young baseline). When OC and JM glomeruli were combined, activated PEC density was 1.49 fold higher in aged mice, but this was not statistically significant (1.88±1.23 vs. 1.26±0.58, p=0.147 aged baseline vs. young baseline).

***PEC activation in FSGS***: In OC glomeruli at D28 of FSGS, activated PEC density increased 3.64 fold in young FSGS mice (1.82±1.45 vs. 0.50±0.23 p<0.05 young FSGS vs. young baseline) and 6.08 fold in aged FSGS mice (9.97±3.71 vs. 1.64±1.29, p<0.0001 aged FSGS vs. aged baseline). In JM glomeruli at D28 of FSGS, a more modest increase of 2.55 fold in young mice (4.97±3.39 vs. 1.95±0.95, p<0.05 young FSGS vs. young baseline) was observed, whereas a 5.62 fold increase was seen in aged FSGS mice (12.43±4.75 vs. 2.21±1.82, p<0.0001 aged FSGS vs. aged baseline). When OC and JM glomeruli were combined, activated PEC density increased 3.14 fold in young FSGS mice (3.96±4.07 vs. 1.26±0.58, p=0.054 young FSGS vs. young baseline), and 6.08 fold in aged FSGS mice (11.43±3.78 vs. 1.88±1.23, p<0.0001 aged FSGS vs. aged baseline).

These results show that at baseline, only OC glomeruli in aged mice had a significantly higher density of activated PECs. As a result of FSGS in both young and aged mice, there was a significant increase in PEC activation. When comparing young FSGS and aged FSGS mice, the density of activated PECs was significantly higher in aged FSGS mice in OC, JM and combined OC and JM glomeruli. Moreover, activated PEC density was most marked in OC glomeruli in both young FSGS and aged FSGS mice when compared to baseline.

### The percentage of total PECs undergoing activation was highest in aged FSGS mice

The results above show that PEC density increased in aged FSGS mice more than young FSGS mice, resulting in similar PEC densities by D28 of FSGS. To further understand if the fraction of total PECs undergoing activation was different between young and aged mice, we next calculated the percentage of total PECs (PAX8^+^) that were activated (PAX8^+^CD44^+^) in OC, JM and combined OC and JM glomeruli of young and aged mice (Figure 3A-C). Table 1 reports the percentage of all PECs that were activated.

***Percentage of PEC activation at baseline***: At baseline in OC glomeruli, the percentage of total PECs that were activated was 3.51 fold higher in aged mice compared to young mice (8.73±1.88% vs. 2.49±0.41%, p<0.01 aged baseline vs. young baseline). However, there was only a 1.32 fold difference in the percentage of activated PECs in JM glomeruli (13.06±3.14% aged vs.9.89±1.4%, p=0.372 aged baseline vs. young baseline). When OC and JM glomeruli were combined, the percentage of PECs that were activated was 1.58 fold higher in aged mice (10.52±2.02% vs. 6.66±0.87%, p=1.00 aged baseline vs. young baseline).

***Percentage of PEC activation in FSGS***: In OC glomeruli at D28 of FSGS, activated PEC percentage increased 2.93 fold in young FSGS mice (7.30±5.47% vs. 2.49±1.16% p<0.05 young FSGS vs. young baseline) and 4.16 fold in aged FSGS mice (36.34±11.84% vs. 8.73±6.51%, p<0.0001 aged FSGS vs. aged baseline). In JM glomeruli at D28 of FSGS, there was a 1.84 fold increase in young mice (18.22±8.61% vs. 9.89±3.95%, p<0.05 young FSGS vs. young baseline). The increase in JM glomeruli in aged FSGS mice was 3.52 fold (46.03±16.00% vs. 13.06±10.87%, p<0.0001 aged FSGS vs. aged baseline). When OC and JM glomeruli were combined, activated PEC percentage increased 1.96 fold in young FSGS mice (13.06±6.31% vs. 6.66±2.47% p<0.01young FSGS vs. young baseline), and 3.89 fold in aged FSGS mice (40.91±13.05% vs. 13.06±10.87%, p<0.0001 aged FSGS vs. aged baseline) (Table 1, Figure 3).

These results show that the percentage of total PECs undergoing activation was substantially higher in aged FSGS mice compared to young FSGS mice in OC, JM and combined glomeruli.

### The migration of activated PECs to the glomerular tuft was higher in aged mice with FSGS

**Figure 4:**
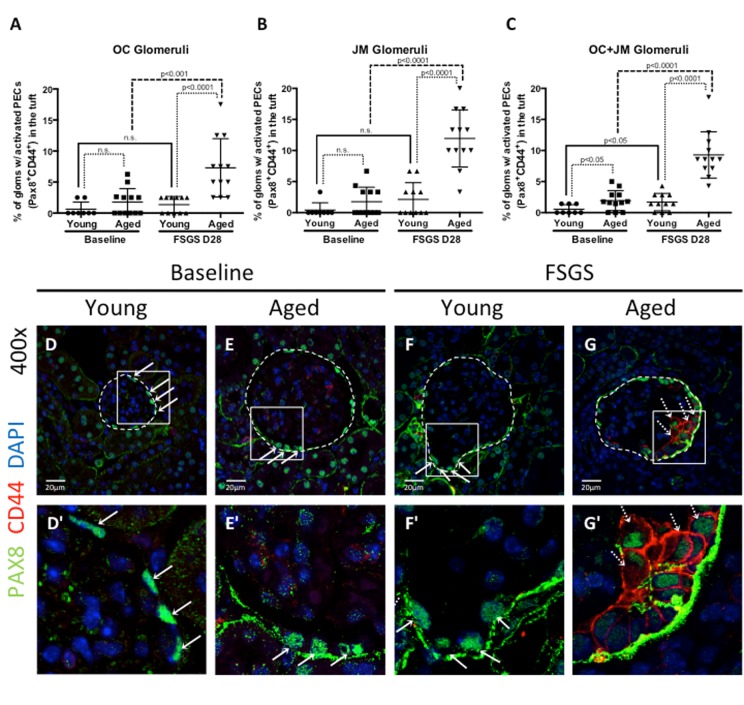
Activated PECs migrated from Bowman's capsule to the glomerular tuft in FSGS (**A-C**) Quantitation showing the percentage of glomeruli with activated PECs (PAX8^+^CD44^+^) on the glomerular tuft. Aged FSGS mice had the highest percentage of activated PECs on the tufts of outer cortical (OC) (**A**), juxta-medullary (**B**) and combined OC and JM (**C**) glomeruli. (**D-G**) Representative images of Pax8 and CD44 staining on tuft. Images of glomeruli (400x) taken by confocal microscopy, showing staining for PAX8 (PEC marker, green, solid arrows), CD44 (activation marker, red, dashed arrows) and DAPI (nuclei, blue). Glomeruli are marked by the dashed line. (**D’-G’**) Higher power images of the area demarcated by the solid square shown above. PAX8 staining was detected along Bowman's capsule in young baseline (**D, D’**) and aged baseline (**E, E’**) mice, but not in the glomerular tuft. (**F, F’**) In young FSGS mice, activated PECs were not readily detected on glomerular tufts. (**G, G’**) Activated PECs were detected on the glomerular tuft of aged FSGS mice. These results show that activated PECs were detected on the tuft of a subset of aged FSGS glomeruli.

Studies have shown that a subset of activated PECs migrate from Bowman's capsule to the glomerular tuft in FSGS, which is responsible in part for segmental glomerulosclerosis [20, 23, 25-28]. The migration of activated PECs to the glomerular tuft was defined as the presence of double positive Pax8^+^CD44^+^ cells on the glomerular tuft (see Figure 4 for examples). The percentage of glomeruli with Pax8^+^CD44^+^ cells on their glomerular tufts was calculated (Figure 4 A-C).

***Migration at baseline***: At baseline, there was no difference between the percentages of OC glomeruli with activated PECs on the tuft between young and aged mice (0.63±0.41% vs. 1.78±0.63%, p=0.140 young baseline vs. aged baseline). Likewise, there was no difference in JM glomeruli (0.42±0.42% vs. 1.75±0.67%, p=0.110 young baseline vs. aged baseline). However, albeit very low numbers, there was an increase when OC and JM glomeruli were combined (0.54±0.26% vs. 1.93±0.46%, p<0.05 young baseline vs. aged baseline).

***Migration in FSGS***: In young FSGS mice, the percentage of glomeruli with activated PECs on their tufts did not increase significantly from young baseline in OC glomeruli (from 0.63±1.16% to 1.36±1.31%, p=0.212 young baseline vs. young FSGS), nor in JM glomeruli (from 0.42±1.18% to 2.12±2.70%, p=0.082 young baseline vs. young FSGS). However, when OC and JM glomeruli were combined, the percentage with activated PECs on their tufts increased significantly in young FSGS mice (1.69±1.40% vs. 0.54±0.74%, p<0.05 young FSGS vs. young baseline). In aged FSGS mice, the percentage of glomeruli with activated PECs on their tufts increased 3.1 fold in OC glomeruli (from 1.78±2.17% to 7.29±4.70%, p<0.01 aged baseline vs. aged FSGS), 6.82 fold in JM glomeruli (from 1.75±2.33% to 11.94±4.60%, p<0.0001 aged baseline vs. aged FSGS), and 4.81 fold when OC and JM glomeruli were combined (from 1.93±1.16% to 9.29±3.73%, p<0.0001 aged baseline vs. aged FSGS). When comparing young and aged mice with FSGS, the percentage of glomeruli with activated PECs on their tufts was higher in aged FSGS mice by 5.35 fold in OC glomeruli, 5.63 fold in JM glomeruli and 5.50 fold in combined OC and JM glomeruli.

These results show that the percentage of OC, JM and OC and JM glomeruli combined in which activated PECs migrated from Bowman's capsule to the glomerular tuft was significantly higher in aged FSGS mice compared to young FSGS mice.

### Active ERK was highest in aged FSGS mice

**Figure 5:**
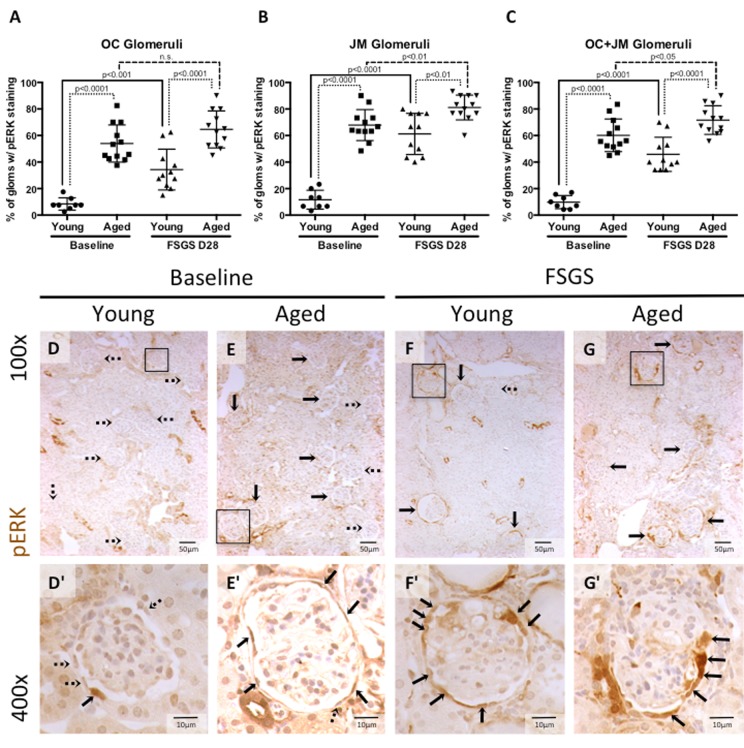
Percentage of glomeruli with phosphorylated-ERK stained PECs was highest in aged mice with FSGS (**A-C**) Quantitation showing the percentage of glomeruli with pERK staining of PECs along Bowman's capsule. Aged baseline mice and aged mice with FSGS had higher percentages of glomeruli with pERK staining along Bowman's capsule when compared to their respective young baseline and young FSGS mice in outer cortical glomeruli (OC) (**A**), juxta-medullary glomeruli (**B**) and combined OC and JM glomeruli (**C**). Overall, aged FSGS mice had the highest percentage of glomerular with pERK staining (**C**). (**D-G**) Representative images of pERK staining along Bowman's capsule. Representative images of glomeruli at 100x magnification, with 400x magnifications shown in D’-G’ of the glomeruli marked by solid black square. Dashed arrows indicate pERK negative and solid arrows indicated pERK positive glomeruli (100x) and PECs (400x).

An age dependent increase in active ERK1/2 in aortic smooth muscle cells has been shown to coincide with increased CD44 and cell motility [29]. Similarly, we have recently reported that active ERK1/2 (phospho-ERK), is an important regulator for the increase of CD44 in PECs themselves [30]. Therefore, staining for phosphorylated ERK was performed to explore ERK1/2 activation as a potential mechanism for higher CD44 and motility of PECs in aged FSGS mice. The percentage of glomeruli with phosphorylated ERK (pERK) staining along Bowman's capsule was quantified and is shown in Figure 5.

***pERK at baseline***: The percentage of glomeruli with pERK staining was significantly higher in aged baseline versus young baseline mice, in the OC, JM, and when the OC and JM glomeruli were combined (8.44±4.2% vs. 53.98±14.00%, 11.67±7.13% vs. 67.92±11.67%, and 9.82±4.97% vs. 60.15±12.31%, respectively, all p<0.001 young baseline vs. aged baseline).

***pERK in FSGS***: The percentage of glomeruli with pERK staining along Bowman's capsule was also signi-ficantly higher in aged FSGS versus young FSGS mice, in the OC (34.32±15.37% vs. 64.58±13.93%, p<0.0001 young FSGS vs. aged FSGS), JM (61.21±15.51% vs. 81.11±9.36%, p<0.01young FSGS vs. aged FSGS), and when the OC and JM glomeruli were combined (45.84±12.88% vs.71.67±10.76%, p<0.0001 young FSGS vs. aged FSGS).

These results show that the percentage of glomeruli with active ERK1/2 increased in both young and aged mice with FSGS, with the highest percentage of glomeruli staining in aged mice with FSGS. Similar to the studies described above [29, 30], this increase correlates with the increase in CD44 positive PECs along Bowman's capsule and the increase in activated PECs that migrated to the glomerular tuft.

### EMT marker expression increased with age and FSGS

**Figure 6:**
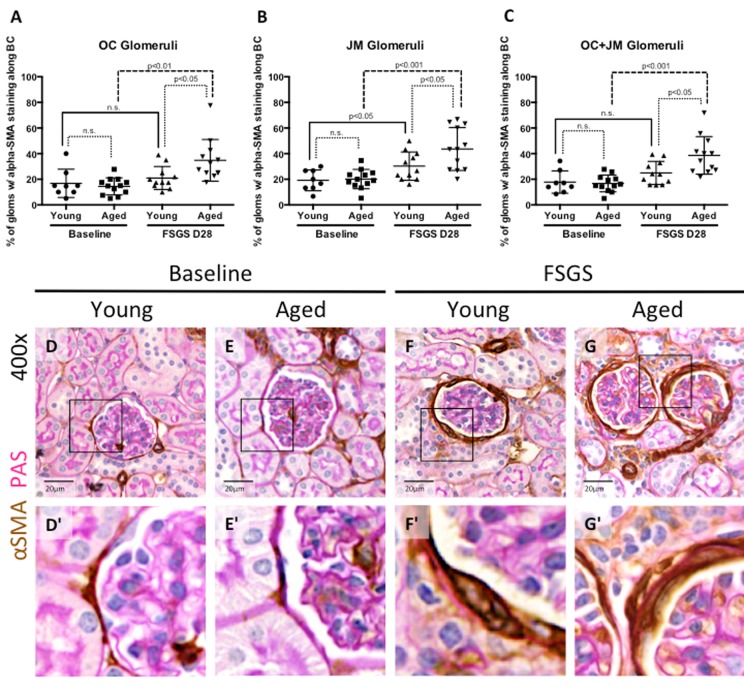
EMT marker staining along Bowman's capsule was highest in aged FSGS mice (**A-C**) Quantitation showing the percentage of glomeruli with α-SMA (EMT marker) staining along BC. There was no significant difference in young and aged mice at baseline in OC (**A**), JM (**B**), or combined (**C**) glomeruli. In OC (**A**), JM (**B**), and combined (**C**) glomeruli, α-SMA staining increased with disease in aged animals, while only in the JM (B) was α-SMA staining significantly increased in young mice, likely due to large variation within the sample groups. (**D-G**) Representative images of glomeruli with alpha-SMA staining along BC taken at 40x. Frequency and intensity α-SMA staining increased with disease (**D** vs. **F**, **E** vs. **G**), despite similar levels between young and aged animals at baseline (**D** vs. **E**). (**D’-G’**) Higher power images of the area demarcated by the solid square shown above, emphasizing the increase in α-SMA staining of cells along BC (**F’**, **G’**).

Studies have shown that changing an epithelial cell's fate to a mesenchymal phenotype i.e. epithelial-to-mesenchymal transition (EMT), is typically accompanied by injurious consequences such as matrix accumulation [31-33]. Staining was performed for the EMT marker α-SMA (Figures 6D-G, D’-G’), previously described in PECs in disease [2, 34-36], and was quantitated and expressed as the percentage of glomeruli with α-SMA staining by kidney region (OC and JM glomeruli), and within these glomeruli, by the intra-glomerular staining pattern along Bowman's capsule (BC), in the tuft only, and along both BC and in the tuft.

***α-SMA staining in OC glomeruli*** (Figure 6A): In young and aged mice, α-SMA staining was mostly confined to cells lining Bowman's capsule. Within OC glomeruli, there was no difference in the percentage of glomeruli with α-SMA staining in young baseline and aged baseline mice (16.83±11.01% vs. 14.47±6.67%, p=0.599, young baseline vs. aged baseline). The percentage of OC glomeruli with α-SMA staining did not increase in young mice with FSGS (21.00±8.97% vs. 16.83±11.01%, p=0.395 young FSGS vs. young baseline). In contrast, the percentage of OC glomeruli with α-SMA staining increased significantly in aged FSGS mice (34.79±16.29% vs. 14.47±6.67%, p<0.01 aged FSGS vs. aged baseline). OC glomeruli with α-SMA staining was higher in aged FSGS mice than young FSGS mice (34.79±16.29% vs. 21.00±8.97%, p<0.05 aged FSGS vs. young FSGS).

***α-SMA staining in JM glomeruli*** (Figure 6B): Within JM glomeruli, there were no significant differences in the percentage of glomeruli with α-SMA staining between young baseline and aged baseline mice (20.15±7.52% vs. 19.17±8.12%, p=0.789 aged baseline vs. young baseline). JM glomeruli with α-SMA staining increased in both young FSGS mice (30.25±11.07% vs. 19.17±8.12%, p<0.05 young FSGS vs. young baseline) and aged FSGS mice (43.66±16.74% vs. 20.15±7.52%, p<0.001 aged FSGS vs. aged baseline). Lastly, aged mice with FSGS also had a significantly higher percentage of JM glomeruli with α-SMA staining when compared to young mice with FSGS (43.66±15.92% vs. 30.25±11.07%, p<0.05 aged FSGS vs. young FSGS).

***α-SMA staining in OC and JM glomeruli*** (Figure 6C): Finally, when glomeruli of the OC and JM were combined, there were no significant differences in the percentages of combined OC and JM glomeruli staining for α-SMA between young baseline and aged baseline mice (17.83±3.08% vs. 16.75±1.89%, p=0.770 young baseline vs. aged baseline). The percentage of combined OC and JM glomeruli with α-SMA staining did not increase significantly in young FSGS mice (24.96±9.00% vs. 17.83±8.72%, p=0.102 young FSGS vs. young baseline) but did increase significantly in aged FSGS mice (38.62±14.70% vs. 16.75±6.54%, p<0.001 aged FSGS vs. aged baseline). The percentage of combined OC and JM glomeruli with α-SMA staining was higher in aged FSGS mice (38.62±14.70% vs. 24.96±9.00%, p<0.05 aged FSGS vs. young FSGS). Altogether, these data suggest that the EMT marker α-SMA was increased in PECs along Bowman's capsule in young and aged FSGS mice, but that the extent of α-SMA staining was higher in OC and JM glomeruli of aged FSGS mice.

### Differential intra-glomerular collagen IV staining in young and aged mice with FSGS in glomeruli of the outer cortex (OC) and juxta-medulla (JM)

**Figure 7:**
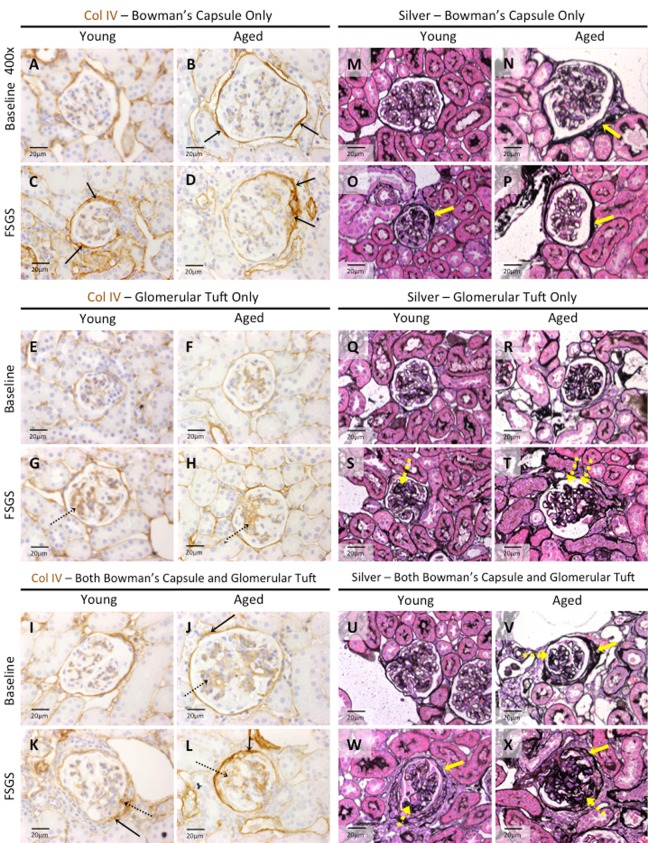
Extracellular matrix accumulation was higher in Bowman's capsule of aged FSGS mice (**A-L**) *Collagen IV (Col IV) staining*. Representative images taken at 40x of Col IV staining (brown color) along Bowman's capsule only (**A-D**, solid arrows), glomerular tuft only (**E-H,** dashed arrows), or along Bowman's capsule and the glomerular tuft (**I-L,** solid and dashed arrows respectively). (**M-X**) Jones’(Silver) staining. Representative images taken at 40x of Jones’ basement membrane staining along BC only (**M-P**, yellow solid arrow), in the glomerular tuft only (**Q-T**, dashed yellow arrow), and both along BC and in the tuft (**U-X**, solid and dashed yellow arrows respectively) confirmed the staining patterns of Col IV.

Because of the different responses in both types of glomerular epithelial cells between young and aged mice in the same model of FSGS, we next examined extracellular matrix accumulation, by staining for collagen IV (Col IV) (Figure 7A-L), and Jones’ basement membrane stain (methenamine silver-Periodic acid Schiff stain, Silver Stain) [37, 38] (Figure 7M-X). Table 2 shows semi-quantitation for Col IV staining performed with two specific questions in mind: are there differences between OC, JM and total glomeruli that stain for Col IV, and within OC and JM glomeruli, are there differences between intra-glomerular staining patterns?

**Table 2:** Percentage of glomeruli with Col IV staining

		Young			Aged			Fold increase and significance in aged mice compared to young mice		
		OC	JM	OC+JM	OC	JM	OC+JM	OC	JM	OC+JM
**% glomeruli with no Col IV staining**	Baseline	83.75±4.63	69.17±11.51	77.50±6.42	42.62±10.83	29.68±10.38	38.07±10.48			
	FSGS D28	47.67±24.92	20.61±12.19	36.07±17.68	35.52±16.94	12.22±11.92	23.87±13.69			
**% glomeruli with Col IV staining along BC only**	Baseline	15.00±4.23	29.58±12.14	21.25±6.54	49.36±10.99	55.77±13.10	51.37±10.94	3.29 fold p<0.0001	1.89 fold p<0.001	2.42 fold p<0.0001
	FSGS D28	25.84±10.98	23.64±10.80	24.90±7.89	43.67±12.49	43.61±11.05	43.65±10.37	1.69 fold p<0.01	1.84 fold p<0.001	1.75 fold p<0.0001
	Fold increase and significance in FSGS from baseline	1.72 fold p<0.05	0.80 fold p=0.288	1.17 fold p=0.288	0.89 fold p=0.249	0.78 fold p<0.05	0.85 fold p=0.09			
**% glomeruli with Col IV staining in the tuft only**	Baseline	0.94±1.29	1.25±1.73	1.07±1.01	3.23±1.89	2.24±3.29	3.00±2.06	3.44 fold p<0.01	1.79 fold p=0.391	2.80 fold p<0.05
	FSGS D28	11.78±10.24	11.82±6.21	11.80±7.84	10.97±5.90	7.22±6.79	9.36±5.28	0.93 fold p=0.821	0.61 fold p=0.105	0.79 fold p=0.398
	Fold increase and significance in FSGS from baseline	12.53 fold p<0.01	9.46 fold p<0.001	11.03 fold p<0.01	3.4 fold p<0.001	3.22 fold p<0.05	3.12 fold p<0.01			
**% glomeruli with Col IV staining along both BC and in the tuft**	Baseline	0.31±0.88	0.00±0.00	0.18±0.51	4.79±6.07	12.30±8.32	7.56±7.07	15.45 fold p<0.05	*young=0 p<0.001	42 fold p<0.01
	FSGS D28	14.71±10.73	43.94±14.21	27.23±9.70	12.84±8.18	36.94±14.17	23.13±9.50	0.87 fold p=0.645	0.84 fold p=0.251	0.85 fold p=0.318
	Fold increase and significance in FSGS from baseline	47.45 fold p<0.01	*baseline=0 p<0.0001	151.28 fold p<0.0001	2.68 fold p<0.05	3.00 fold p<0.0001	3.06 fold p<0.001			
**% glomeruli with Col IV staining by combining BC only, tuft only, BC and tuft**	Baseline	16.25±4.63	30.83±11.51	22.50±6.42	57.38±10.83	70.32±10.38	61.93±10.48	3.53 fold p<0.0001	2.28 fold p<0.0001	2.75 fold p<0.0001
	FSGS D28	52.33±24.92	79.39±12.19	63.93±17.68	67.48±16.94	87.78±11.92	76.13±13.69	1.29 fold p=0.109	1.11 fold p=0.111	1.19 fold p=0.082
	Fold increase and significance in FSGS from baseline	3.22 fold p<0.001	2.58 fold p<0.0001	2.84 fold p<0.0001	1.18 fold p=0.098	1.25 fold p<0.001	1.23 fold p<0.01			

In young baseline mice, Col IV staining was not detected in 84% of OC glomeruli, 69% of JM glomeruli and 78% in combined OC and JM glomeruli (Table 2). This contrasted from aged baseline mice, where Col IV staining was not detected in just 43% of OC glomeruli, 30% of JM glomeruli and 38% of combined OC and JM glomeruli (Table 2). Thus, Col IV staining was present in significantly more glomeruli in both kidney compartments in aged baseline mice compared to young baseline mice.

#### Col IV staining along Bowman's capsule only

***At baseline***: The percentage of OC glomeruli staining for Col IV only along Bowman's capsule was 3.29 fold higher in aged baseline mice than young baseline mice (49.36±10.99% vs. 15.00±4.23%, p<0.0001 aged baseline vs. young baseline). The percentage of JM glomeruli staining for Col IV only along Bowman's capsule was 1.89 fold higher in aged baseline mice (55.77±13.10% vs. 29.58±12.14%, p<0.001aged baseline vs. young baseline). Thus, when OC and JM glomeruli were combined, baseline aged mice had a 2.42 fold higher percentage of glomeruli with Col IV along Bow- man's capsule only (51.37±10.94% vs. 21.25± 6.54%, p<0.0001 aged baseline vs. young baseline).

***In FSGS***: In young FSGS mice, there was a small but significant 1.72 fold increase in the percentage of OC glomeruli with Col IV along Bowman's capsule only (25.84±10.98% vs. 15.00±4.23%, p<0.05 young FSGS vs. young baseline). In contrast, in OC glomeruli, Col IV staining only along Bowman's capsule did not increase in aged FSGS mice (43.67±12.49% vs. 49.36±10.99%, p=0.249 aged FSGS vs. aged baseline), but the percentage of glomeruli with staining in OC glomeruli was 1.69 fold higher in aged FSGS mice compared to young FSGS mice (43.67±12.49% vs. 25.84±10.98%, p<0.01 aged FSGS vs. young FSGS).

The percentages of glomeruli with or without Col IV staining are given for young and aged mice. Labeled bold, the top row shows the percentages of glomeruli without Col IV staining, the middle three rows show the percentages of glomeruli with staining only at specific regions (along BC only, in the tuft only, and along BC and in the tuft). The final row is a total of the middle three rows, representing the total percentage of glomeruli with Col IV staining. For both young and aged mice, values are given for OC, JM, and combined OC and JM. One important note is that the OC+JM value is not necessarily the average of the OC and JM region values. Rather, the combined OC+JM value is the average of 40 OC and 30 JM (when possible) glomeruli together as roughly 70 representative glomeruli of the total section. Data are represented as mean±SD. Fold increases (≥1) from baseline to FSGS, and also between young and aged mice at baseline and FSGS, are given below and to the right side of the table respectively. A fold increase of less than 1 implies a fold decrease. Significance is given in the same box as fold change. OC - outer cortex; JM - juxta-medullary; SD - standard deviation.

In JM glomeruli, Col IV staining along Bowman's capsule only did not increase in either young FSGS or aged FSGS mice (Table 2). When combining OC and JM glomeruli, Col IV along Bowman's capsule only was almost 2 fold higher in aged FSGS mice (43.65±10.37% vs. 24.90±7.89%, p<0.0001, aged FSGS vs. young FSGS).

These results show Col IV staining along Bowman's capsule only was higher in aged baseline mice compared to young baseline mice, and that this difference persisted in FSGS.

#### Col IV staining in glomerular tuft only

***At baseline***: Although staining Col IV staining in tuft only was low in baseline mice, the percentage of OC glomeruli was 3.44 fold higher in aged baseline mice compared to young baseline (3.23±1.89% vs. 0.94±1.29%, p<0.01 aged baseline vs. young baseline), but there were no differences in JM glomeruli. However, when the OC and JM glomeruli were combined, the percentage with Col IV staining in tuft only was 2.8 fold higher in aged baseline mice (3.00±2.06% vs. 1.07±1.01%, p<0.05 aged baseline vs. young baseline).

***In FSGS***: The percentage of OC glomeruli with Col IV staining increased 12.53 fold in young FSGS mice (11.78±10.24% vs. 0.94±1.29%, p<0.01 young FSGS vs. young baseline), and 3.4 fold in aged FSGS mice (10.97±5.90% vs. 3.23±1.89%, p<0.001 aged FSGS vs. aged baseline). There was no statistical difference between OC glomeruli between young and aged FSGS mice.

The percentage of JM glomeruli with Col IV staining in the glomerular tuft only increased 9.46 fold in young FSGS mice (11.82±6.21% vs. 1.25±1.73%, p<0.001 young FSGS vs. young baseline), and 3.22 fold in aged FSGS mice (7.22±6.79% vs. 2.24±3.29%, p<0.05 aged FSGS vs. aged baseline). Because of the high variation in staining between animals within groups, the results in FSGS were not statistically different between young and aged FSGS mice.

When OC and JM glomeruli were combined, there was an 11.03 fold increase in young FSGS mice (11.80±7.84% vs. 1.07±1.01%, p<0.01 young FSGS vs. young baseline) and 3.12 fold increase in aged FSGS mice (9.36±5.28% vs. 3.00±2.06%, p<0.01 aged FSGS vs. aged baseline). However, there was no significant difference between young FSGS and aged FSGS mice.

These results show that at baseline, staining for Col IV in the tuft only was higher only in OC glomeruli of aged mice. In FSGS, the increase from baseline was substantially higher in young mice in OC and JM glomeruli, resulting in no differences in the percentages of OC, JM and combined OC and JM glomeruli with Col IV staining in the glomerular tuft only.

#### Col IV staining along Bowman's capsule and in the glomerular tuft

***At baseline***: The percentage of OC glomeruli with Col IV staining along Bowman's capsule and in the glomerular tuft was 15.45 fold higher in aged baseline mice (4.79±6.07% vs. 0.31±0.88%, p<0.05 aged baseline vs. young baseline). This difference was even more striking in JM glomeruli (12.30±8.32% vs. 0.00±0.00%, p<0.001 aged baseline vs. young baseline), and when OC and JM glomeruli were combined (7.56±7.07% vs. 0.18±0.51%, p<0.01aged baseline vs. young baseline).

***In FSGS***: The percentage of OC glomeruli with Col IV staining along Bowman's capsule and in the glomerular tuft increased 47.45 fold in young FSGS mice (14.71±10.73% vs. 0.31±0.88%, p<0.01 young FSGS vs. young baseline), and 2.68 fold in aged FSGS mice (12.84±8.18% vs. 4.79±6.07%, p<0.05 aged FSGS vs. aged baseline). Consequently, there was no difference in OC glomeruli between young FSGS and aged FSGS mice.

The percentage of JM glomeruli with Col IV staining along Bowman's capsule and in the glomerular tuft increased markedly in young FSGS mice (43.94±14.21% vs. 0.00±0.00%, p<0.0001 young FSGS vs. young baseline), and 3 fold in aged FSGS mice (36.94±14.17% vs. 12.30±8.32%, p<0.0001 aged FSGS vs. aged baseline). However, there was no statistical difference in JM glomeruli between young FSGS and aged FSGS mice. Similarly, there was no statistical difference in combined OC and JM glomeruli were combined between young FSGS and aged FSGS mice.

Similar results were obtained for Jones’ basement membrane staining.

## DISCUSSION

Focal segmental glomerulosclerosis (FSGS), a leading cause of primary glomerular diseases in the USA [39], is a major cause of chronic and end-stage kidney disease [40, 41]. The incidence and prevalence of FSGS may be increasing or becoming more frequently found within in the aging population [42, 43]. Moreover, although numerous variables impact FSGS outcomes and disease course, a higher age typically portends a poorer prognosis in glomerular diseases[44].

FSGS is characterized by initial injury to the glomerular epithelial cell called the podocyte, with secondary activation in the neighboring glomerular parietal epithelial cells (PEC) [20, 22-24, 27]. However, the impact of age on the response of both glomerular epithelial cell subtypes in FSGS are not well understood, nor is it known if advanced age alters the responses to injury of these cells based on their location in the outer cortex (OC) or juxta-medulla (JM). Using a pre-clinical model of FSGS, the results of the current study shows that compared to young FSGS mice, aged FSGS mice have lower podocyte density, higher parietal epithelial cell (PEC) activation, migration and EMT, accompanied by increased collagen IV staining along Bowman's capsule.

In the current study, we compared 3m-old young baseline mice (≈ human age 20 years) to 27m-old aged baseline mice (≈ human age 78 years), and then 28 days following the induction of experimental FSGS, young FSGS mice were compared to aged FSGS mice [16]. We acknowledge the fact that there is substantial variation between aged mouse strains and between individual aged mice [21], which is not unlike the variation that exists within the human population. To mitigate these differences, and to best reflect the human aspect behind pursuing such studies, we used an in house bred F1 generation of a mixed mouse strain for both the young and old mice that were within 3 generations of each other. We also included both male and female mice in this study. Our results show no significant differences between males and females in any of the study parameters measured.

To better understand if there were substantive differences between different regions of the kidney, we analyzed data separately within each group, divided into glomeruli of the outer cortex (OC), glomeruli of the juxta-medulla (JM), and when glomeruli from both OC and JM regions were combined. It is well established that podocyte depletion underlies glomerular scarring in secondary forms of experimental and human FSGS [15, 45-48]. The experimental FSGS model used in this study is typified by acute podocyte depletion by D3 that persists until D14, followed by partial podocyte replacement at D28 [17, 19, 20, 49]. Several interesting podocyte results were noted. First, podocyte density was lower in aged baseline mice compared to young baseline mice. This was expected based on reports by our group [2, 50] and others [15, 51]. Second, the extent of podocyte depletion from their respective baselines (pre-disease) was significantly higher in young FSGS mice compared to aged FSGS mice. Third, the magnitude of podocyte depletion was more marked in both OC and JM glomeruli of young FSGS mice. Fourth, despite these changes, the podocyte density at D28 was lower in aged FSGS mice compared to young FSGS mice in OC, JM and combined OC and JM glomeruli. We interpret the more severe depletion of podocytes in young FSGS mice to reflect a higher overall podocyte density from the outset, and the resultant lower podocyte density in aged FSGS mice to reflect a lower baseline podocyte density in aged mice prior to disease.

The results showed that PEC density, defined as the number of PAX8^+^ cells per length of Bowman's capsule, was higher in OC glomeruli of both young FSGS and aged FSGS mice compared to their baselines. PEC density increased in JM glomeruli of aged FSGS mice, but not in JM glomeruli of young FSGS mice. To better understand the biological nature of the increase in PECs, we co-stained for CD44, because seminal studies by Smeets and Moeller in experimental and human FSGS showed activation of PECs, defined as the de novo staining for CD44 in PECs [23-25]. Indeed, we and others have shown that PEC activation accompanies changes to the fate of PECs including pro-fibrogenic and pro-migratory phenotypes [23-25]. Moreover, we have reported PEC activation in the FSGS model used in the current studies in young adult mice [20], and have also reported that in mice with advanced age, but without disease, CD44 staining is higher in PECs compared to normal young mice [2]. Finally, we recently showed for the first time that CD44 is biologically functional in PECs underlying their pro-fibrotic and pro-migratory responses to injury [30]. The second major finding in the current study was that the density of activated PECs, defined as the number of PAX8^+^CD44^+^ stained cells per length of Bowman's capsule, was significantly higher in aged FSGS mice compared to young FSGS mice in glomeruli of the OC (5 fold higher), JM (2.4 fold) and when OC and JM are combined (2.75 fold). Moreover, we show that active phosphorylated form of ERK is also higher in aged FSGS mice, which is likely biological important because we have reported that this active form of ERK is a critical signaling pathway underlying an increase in CD44 in PECs [30].

We next analyzed what percentage of PECs (PAX8^+^) were activated (defined as PAX8^+^ CD44^+^) in glomeruli of the OC and JM. In OC glomeruli, 7.30±5.47% and 36.34±11.84% of all PECs were activated in young FSGS and aged FSGS mice respectively; in JM glomeruli, 18.22±8.61% and 46.03±16.00% of all PECs were activated in young FSGS and aged FSGS mice respectively, resulting in 13.06±6.31% and 40.91±13.05% of all PECs activated when the OC and JM glomeruli were combined in young FSGS and aged FSGS mice respectively. Smeets et al. [23] and our group [20] have shown that a subset of activated PECs migrate from Bowman's capsule to the glomerular tuft in FSGS. In the current study the percentage of activated PECs that migrated to the glomerular tuft was significantly higher in diseased aged mice in OC, JM and combined OC and JM glomeruli.

Taken together, the PEC data might be interpreted as follows: first, although overall PEC density was lower in aged baseline mice compared to young baseline mice, the PEC density increase in response to FSGS was higher in aged mice, so that total PEC density was similar in disease between young and aged FSGS mice. Second, the density of PECs undergoing activation was higher in aged FSGS mice compared to young FSGS mice in OC, JM and combined OC and JM glomeruli. Third, the percentage of PECs that underwent activation (activated PECs) was substantially higher in aged FSGS mice in OC, JM and combined OC and JM glomeruli. Fourth, the migration of activated PECs to glomerular tufts is higher in aged FSGS mice than young FSGS mice.

Studies have reported that in addition to undergoing activation in FSGS, a subset of PECs undergo fate switches from their epithelial phenotype to a mesenchymal phenotype, known as epithelial-to-mesenchymal transition (EMT). Indeed, we have previously shown PEC EMT in this model in young mice [35]. Using the EMT marker α-SMA, the third major finding in the current study was that in FSGS, PEC EMT was significantly higher in aged FSGS mice compared to young FSGS mice.

Thus, the results of this study showed that in experimental FSGS, aged mice had a lower podocyte density, higher PEC activation, migration and EMT. We next measured collagen IV (Col IV) staining as a marker of extracellular matrix accumulation in OC and JM glomeruli. Several key observations were noted in FSGS mice: (i) although Col IV staining along a Bowman's capsule only distribution increased in young FSGS mice compared to young baseline mice, the percentage of glomeruli with this staining pattern was significantly higher in aged mice in OC, JM and combined OC and JM glomeruli. This almost certainly was due to a markedly higher degree of Col IV staining along Bowman's capsule prior to disease in aged baseline mice. (ii) Despite a substantially greater fold increase in Col IV staining in the glomerular tuft in young FSGS mice from baseline compared to the increase in aged FSGS mice, there were no differences in Col IV staining between young FSGS and aged FSGS mice in OC, JM and combined OC and JM glomeruli. We speculate that there is a maximal level to which Col IV staining can increase in injured glomeruli. (iii) Although the increase in Col IV staining in both Bowman's capsule and the glomerular tuft increased markedly more from baseline in young FSGS mice compared to aged FSGS mice, the percentage of glomeruli with Col IV staining in this distribution was similar between young and aged FSGS mice in OC, JM and combined OC and JM glomeruli. (iv) In FSGS, Col IV staining in glomerular tufts with or without accom-panying Col IV staining along Bowman's capsule was higher in JM glomeruli of both young FSGS and aged FSGS mice. Taken together, the fourth major finding was that in FSGS, the percentage of glomeruli with collagen IV staining along Bowman's capsule was higher in aged FSGS mice, but collagen IV staining was similar in the glomerular tufts of young and aged FSGS mice.

The PEC is often a secondary target of damage following injury to other glomerular cell types or the glomerular basement membrane [52, 53]. The triggers and mechanisms responsible for this are still unclear. In our recent publication [54], we found evidence of mitochondrial damage, increased reactive oxygen species (nicotinamide adenine dinucleotide phosphate oxidase 4 staining) and senescence (p16 and senescence-associated-b-galactosidase staining) in PECs of aged mice. Interestingly, these effects were somewhat reversible when Elamipretide (SS-31), a mitochondria-targeted tetra- peptide was administered. The pathways highlighted likely prime the PEC to be particularly prone to damage in aging following other forms of glomerular injury. In this study, we showed that following primary damage to the podocyte, aged PECs were particularly susceptible to secondary injury. An obvious therapeutic would be to administer Elamipretide to aged mice and subject them to primary podocyte damage and see if the effects seen in this study are abrogated.

We recognize several limitations to these studies. First, although largely descriptive in nature, they do provide important insights into the different responses of glomerular epithelial cells in FSGS. Second, no direct mechanism is shown. We recently reported that an increase in active ERK is a critical signaling pathway for increased CD44 and motility in PECs [30]. Similarly, increases in active ERK, CD44 and cell motility have been shown in aortic smooth muscle cells with age, leading to intima thickening during vascular neointima formation [29]. In the current study, we provide data showing a significant increase in both pERK and CD44 in aged PECs, leading us to consider this axis as a possible explanation for the changes in PECs in aged FSGS mice compared to young FSGS mice. Third, although albuminuria was measured, glomerular filtration rate was not. While this should have ideally been performed, such measures are oftentimes unreliable in aged mice, as they are in humans. Beyond albuminuria, no intermediary time points were studied, leading one to make assessments and thus conclusions based on a single time point following FSGS. Because of the changes to glomerular size with age, the length of Bowman's capsule and the volume of the glomerular tuft were measured to calculate densities of critical PEC and podocyte endpoints respectively. We also recognize that due to difficulties inherent to aging studies, such as the great variation in aging characteristics of individual animals, this consequently results in a high variance within many of the parameters that were quantified. Fourth, we elected to dose the anti-podocyte antibody by body weight, as opposed to a fixed dose for both young and old mice. Because aged mice were typically 40% heavier than younger mice, younger mice would have received 40% more of the disease-inducing antibody. We do not believe that this accounted for differences between young and aged mice, because following antibody administration to deplete podocytes, the abrupt decrease in podocyte number from baseline was significantly greater in young FSGS mice compared to aged mice. Furthermore, staining for antibody binding in the glomerulus was similar in young and aged mice. Moreover, we performed a pilot study in young and aged mice using a fixed dose of disease inducing antibody in which the dose of the antibody was approximately midway between the weights of young and aged mice. Our results showed that the younger mice lost more weight, ate less and had more ruffled hair compared to their aged counterparts. These clinical changes compelled us to use a weight adjusted dosing schema, which negated the abovementioned side effects. However, we recognize that the slight differences in dosing of the antibody needs to be considered when evaluating the results.

In summary, following similar binding of the cytopathic anti-podocyte antibody in young and aged mice to induce podocyte depletion, the responses by podocytes and PECs differed between young and aged mice with disease. Overall podocyte density was lower in aged FSGS mice compared to young FSGS mice, largely due to their baseline density being lower. Furthermore, the percentage of overall PECs that underwent activation and EMT in both outer cortical and juxta-medullary glomeruli was significantly higher in aged FSGS mice. Our results hint at pERK as a potential mechanism, an association that needs further exploration. These results might shape future studies in which specific pathways might be targeted differently in the young and aged with disease.

## METHODS

### Study animals

All mice studied were of the same mixed background strain (F1 offspring from a B10 X C3H F1 female crossed with a B6 male) bred in house, and included both male and female animals. Young adults, 3 months of age (n=8), served as baseline controls. Another group of mice (n=12), of the same age, were given experimental FSGS via a cytotoxic anti-glomerular antibody. In another cohort, aged mice, 24 months of age (n=12), first underwent survival kidney biopsies, the tissue from which served as their baseline. Following two weeks of surgical recovery, mice were given experimental FSGS via a cytotoxic anti-glomerular antibody. Mice were housed in the animal care facility at the University of Washington under specific pathogen-free conditions with ad libitum access to chow and water. Animal protocols were approved by the University of Washington Animal Care Committee. At sacrifice, mice were perfused with 10ml ice cold PBS to remove excess red blood cells. Kidneys were bisected, in order for one half to be fixed in 10% neutral buffered formalin (Globe Scientific, Paramus, NJ) at 4°C overnight, then rinsed in 70% ethanol, processed and embedded in paraffin. The other half was fixed for 45 minutes in 4% PFA in PBS (Affymetrix, Santa Clara, CA), washed in 30% sucrose at 4°C overnight, patted dry, rinsed briefly in optimum cutting temperature (OCT) compound (Sakura Finetek, Torrance, CA), embedded in OCT, and frozen in a dry ice 100% ethanol bath. These blocks were then cut into 4μm sections for immunostaining.

### Experimental FSGS with podocyte depletion

The experimental model of FSGS used in this study is characterized by abrupt podocyte depletion, accompanied by glomerulosclerosis, followed by partial podocyte repletion at day 28 [20]. FSGS was induced in mice by two consecutive doses of anti-glomerular antibody, injected IP at 12mg IgG/20g bodyweight, 24hr apart. Specific antibody binding in the glomerulus was verified by staining for sheep IgG.

Podocyte depletion was assessed by p57/PAS staining on paraffin-embedded sections, as previously described [55, 56]. In brief, slides were deparaffinized using Histoclear (National Diagnostics, Atlanta, GA) and rehydrated in a graded series of ethanol baths. Antigen retrieval was performed by boiling the slides in 10mM EDTA pH 8.0. Non-specific antibody binding was blocked using nonfat dry milk for 20 min. Exogenous peroxidase activity was quenched with 3% hydrogen peroxide. Primary rabbit anti-p57 antibody (Santa Cruz Biotechnology, Santa Cruz, CA) was diluted 1:800 in 1% BSA in PBS and was incubated overnight at 4°C, followed by rabbit on rodent HRP-polymer (Biocare Medical, Concord, CA). Staining was visualized with diaminobenzidine (DAB) precipitation (Sigma-Aldrich, St. Louis, MO). PAS counterstaining was performed by washing slides in fresh 0.5% periodic acid (Sigma-Aldrich, St. Louis, MO) for 8 min, followed by double-distilled water for 5 min, then incubating with Schiff's reagent (Sigma-Aldrich) at room temperature for 10 minutes. Slides were then washed in 0.5% sodium metabisulfate (Sigma-Aldrich) and afterwards under running tap water for 5-10 min. Slides were dehydrated in ethanol and mounted with Histomount. Podocyte density was measured with the correction factor (CF) method, as previously reported by Venkatareddy et al. [57]. For p57/PAS staining, and all other stains, glomeruli were separated into outer kidney cortex (OC) and juxta-medullary (JM) regions for quantification purposes as previously described by Roeder et al. [2]. 30 JM and 40 OC glomeruli were counted in each section, and a total sum of OC and JM glomeruli served as a representation of the entire section.

### Assessment of extracellular matrix

Extracellular matrix accumulation was detected on paraffin-embedded tissue by Jones’ basement membrane stain (Silver Stain) performed by the University of Washington Pathology Research Services Laboratory and by staining for matrix protein collagen type IV (Col IV), counterstained with PAS. Staining for Col IV protein was performed with the aforementioned immunoperoxidase protocol, substituting background buster (Accurate Chemical & Scientific, Westbury, NY) for nonfat dry milk in the blocking step. Col IV was scored by classifying glomeruli into four groups: no Col IV staining, staining isolated along Bowman's capsule (BC) only, staining isolated in the glomerular tuft only, or staining both along BC and in the tuft.

### Identification of PECs and ERK activity along Bowman's capsule

To discern PECs, double-immunofluorescence staining was performed for Pax8, which marks PECs [58], and CD44, a marker of activation in profibrotic PECs [24, 27, 59, 60]. Tissue was deparaffinized in Histoclear and rehydrated in a graded series of ethanol baths. Antigen retrieval was performed by boiling in 10mM citric acid buffer pH 6.0. Slides were then blocked with an avidin and biotin blocking kit (Vector Labs, Burlingame, CA). Nonspecific antibody binding was blocked with background buster (Accurate Chemical & Scientific, Westbury, NY). Slides were then incubated with rabbit anti-Pax8 antibody (1:500, Protein Tech Group, Rosemont, IL) overnight at 4°C. Donkey anti-rabbit Alexa Fluor 488 antibody (1:100, Jackson Immunoresearch Laboratories, West Grove, PA) was then applied. Afterwards, slides were blocked with rabbit Fab fragment and secondary goat anti-rabbit Fab fragment (1:25, Jackson Immunoresearch Laboratories, West Grove, PA), and then background buster. Next, rat anti-CD44 (1:10, BD Biosciences, San Jose, CA) was incubated overnight at 4°C. Next, a biotinylated goat anti-rat IgG (1:500, Vector Laboratories, Burlingame, CA) was applied, followed by streptavidin conjugated with Alexa Fluor 594 (1:100, Zymed Laboratories, San Francisco, CA). Slides were mounted in Vectashield (Vector Labs, Burlingame, CA). Positive double staining was defined as a green Pax8^+^ cell, with the physical characteristics of a PEC, co-localized with red CD44^+^ staining. OC and JM glomeruli were quantified for Pax8^+^ stained cells (PEC density) and double positive stained cells (activated PEC density) and their BC length (μm). Double positive cells in the glomerular tuft were also quantified.

To determine ERK activity, staining was performed with rabbit polyclonal antibody to phospho-p44/42 MAPK (1:250, Cell Signaling Technology, Beverly, MA) with the aforementioned immunoperoxidase protocol. The percentage of glomeruli with pERK staining in PECs along Bowman's capsule was quantified in both the OC and JM glomeruli.

### Epithelial to Mesenchymal Transition (EMT) markers

To determine the degree of PEC EMT, α-smooth muscle actin (α-SMA) was performed with PAS counterstaining. Mouse anti-α-SMA (1:10.000, Sigma) antibody was used as we have previously reported [35]. OC and JM glomeruli were quantified for PEC staining along BC. Quantification was performed by counting the percentage of glomeruli with increased α-SMA staining.

### Urine analysis

Urine was collected prior to induction of FSGS at baseline, as well as at D7, D14, D21, and D28 post FSGS in both young and aged cohorts. Urine albumin was determined using a radial immunodiffusion assay (RID) as previously described [61]. Briefly, 2ul of urine was placed into a well cut into a thin layer of agarose made in 0.5 M veronal buffer that incorporated a rabbit anti-mouse albumin antibody (Accurate Chemical, Westbury, NY) and rabbit serum (Pel-Freez, Rogers, AR). A standard curve was prepared from known concentrations of mouse albumin (MP Biomedicals, Irvine, CA) and the halo of precipitation formed around each well on a plate as the urine diffused permitted the determination of urine albumin concentration. Urine creatinine was determined by using a commercially available Creatinine (urinary) Colorimetric Assay Kit (Cayman Chemical, Ann Arbor, MI). A ratio of albumin to creatinine (ACR, μg /mg) was calculated for each sample.

### Microscopy and imaging

Imaging and quantification was performed on a Leica DMI400B microscope and an EVOS FL Cell Imaging System and a Leica TCS SPE II laser scanning confocal microscope.

ImageJ 1.5 (NIH) was used to measure distances and areas.

### Statistical analysis

Statistical analysis was performed using GraphPad Prism 6.0 (La Jolla, CA) and MATLAB (Natick, MA). Student's t-test and 1-way ANOVA with multiple comparisons was applied to compare means of specific group pairs, (Young Baseline vs. Aged Baseline, Young FSGS vs. Aged FSGS, Young Baseline vs. Young FSGS, Aged Baseline vs. Aged FSGS) and p<0.05 was considered statistically significant. MATLAB's Statistics Toolbox was used to compute Gaussian Mixture Model (GMM) graphs. Data are presented as means ± SD.

## SUPPLEMENTARY MATERIALS AND FIGURES


