# Supplementary material for: Compound effects of aging and experimental FSGS on glomerular epithelial cells
Source: Aging (Albany NY). 2017 Feb 17;9(2):524–45. doi: 10.18632/aging.101176 (PMC5361679; doi:10.18632/aging.101176)
Supplement: Supplementary file 1 [file aging-09-524-s001.pdf]

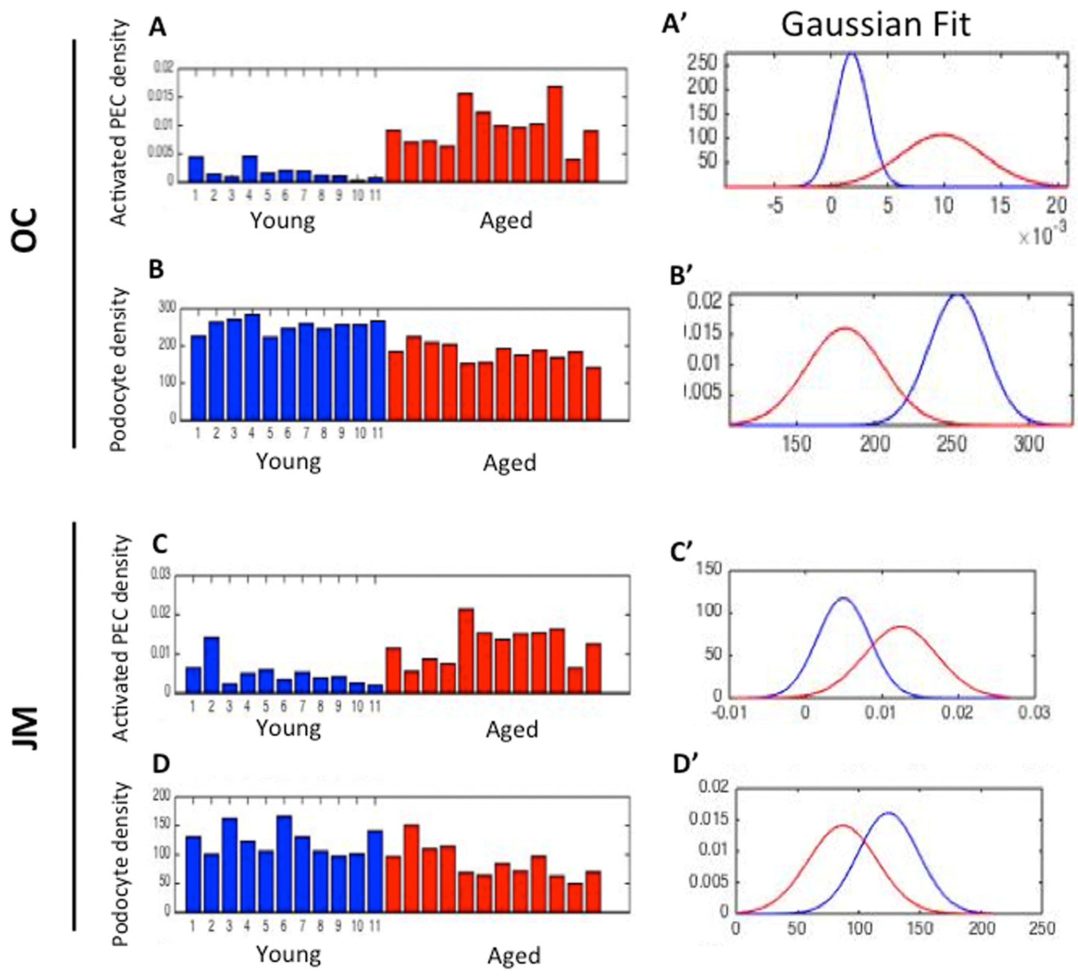

Supplementary Figure 1. Visualizing the differences in activated PEC density and podocyte density between aged and young mice in the OC and the JM at D28 of FSGS, indicating that the OC is the region most affected by age and disease in our model. (**A**) Activated PEC density in the OC for each animal showed a higher number in aged mice, and highlights the high variability in the sampled animals. (**A'**) A Gaussian Mixture Model (GMM), which gives a probabilistic model for representing the presence of subpopulations within an overall population, shows a significant separation in the young versus old animals despite this high variability. (**B**) Podocyte density between aged and young mice in the OC, showing a lower density in aged mice. (**B'**) GMM showing that aged mice have a significantly lower podocyte density. (**C**) Activated PEC density in the JM for each animal showed a higher number in aged mice. (**C'**) A GMM showed the overlap in activated PEC density young and aged animals. (**D**) Podocyte density was lower in the JM of aged mice. (**D'**) GMM showing a significant overlap in podocyte density of young and aged mice in the JM. The GMMs, computed from the MATLAB Statistics Toolbox, show a pronounced statistical difference in aged and young mice in the OC in comparison with the JM.
